# Correlation analysis of serum glycosylphosphatidylinositol mannosyltransferase 1 (GMP1) levels in type 2 diabetes mellitus patients

**DOI:** 10.5937/jomb0-60118

**Published:** 2026-03-17

**Authors:** Xiaolin Yang, Wang Qichang, Wei Chen, Jian Zhao

**Affiliations:** 1 Department of Clinical Laboratory, Affiliated Hospital of Shaoxing University (Shaoxing Municipal Hospital), China; 2 Peking University Shenzhen Hospital, Endocrinology Department, Shenzhen City, China; 3 Affiliated Hospital of Shaoxing University (Shaoxing Municipal Hospital), Department of Clinical Laboratory, Shaoxing City, China

**Keywords:** glycosylphosphatidylinositol mannosyltransferase 1 (GMP1), hypertriglyceridemia (HTG), diabetes disease, glikosilfosfatidilinozitol manoziltransferaza 1 (GMP1), hipertrigliceridemija (HTG), dijabetes

## Abstract

**Background:**

To investigate the serum expression of glycosylphosphatidylinositol mannosyltransferase 1 (GMP1) in type 2 diabetes mellitus (T2DM) patients and its correlation with hypertriglyceridemia (HTG) to shed light on lipid metabolism disorders in T2DM patients.

**Methods:**

A total of 239 patients were included, among whom 92 patients were in the T2DM combined with HTG group and 147 patients were in the T2DM without HTG group. The concentration of the serum GMP1 protein was quantitatively detected via enzyme-linked immunosorbent assay (ELISA). Moreover, the levels of serum triglycerides (TGs) and other related metabolic indicators (such as blood glucose, glycated haemoglobin (HbA1c), total cholesterol, and high/low-density lipoprotein cholesterol) were detected via conventional biochemical methods. To evaluate the potential impact of GMP1 on the occurrence of T2DM combined with HTG.

**Results:**

Both the DM group and the DM + HTG group presented significantly higher serum GMP1 levels (P&lt; 0.01), and the GMP1 level in the DM +HTG group was considerably greater (P&lt; 0.05 or 0.01) than that in the simple DM group. The serum GMP1 levels were significantly greater in T2DM patients than in HTG patients. Serum GMP1 levels (O R = 1.527, 95% CI 1.200-1.943) were determined via binary logistic regression analysis. 95% CI 1.003-1.010) was a separate risk factor for HTG and T2DM. Correlation analysis revealed similar results in patients with T2DM (especially those in the DM +HTG group). Multiple regression analysis further indicated that after controlling for factors such as age, sex, disease duration, BMI, and HbA1c, higher blood GMP1 levels continued to be a predictor or independent factor for patients with T2DM complicated by HTG (P&lt; 0.05).

**Conclusions:**

Serum GMP1 levels are markedly elevated in T2DM patients, especially those with hypertriglyceridemia, and they are independently positively linked with triglycerides.

## Abstract (sr)

**Uvod:**

Cilj e ispita ekspresija serumske glikosilfosfatidilinozitol manoziltransferaze 1 (GMP1) kod pacijenata sa dijabetesom tip 2 (T2DM) i njena korelacija sa hipertrigliceridemijom (HTG), radi boljeg razumevanja poremećaja lipidnog metabolizma kod ovih pacijenata.

**Metode:**

U istraživanje je uključeno ukupno 239 pacijenata, od kojih je 92 pripadalo grupi sa T2DM udruženim sa HTG, dok je 147 pacijenata bilo u grupi sa T2DM bez HTG. Koncentracija serumskog GMP1 proteina je kvantitativno određena metodom enzimski povezanog imunosorbentnog testa (ELISA). Pored toga, nivoi serumskog triglicerida (TG) i drugih relevantnih metaboličkih pokazatelja (npr. glukoza u krvi, glikovani hemoglobin (HbA1c), ukupni holesterol i HDL/LDL holesterol) su određeni standardnim biokemijskim metodama. Procenjivao se potencijalni uticaj GMP1 na pojavu T2DM udruženog sa HTG.

**Rezultati:**

I grupa sa T2DM i grupa sa T2DM +HTG su pokazale značajno povišene serumske nivoe GMP1 (P&lt; 0,01), pri čemu je nivo GMP1 u grupi sa T2DM +HTG bio znatno viši (P&lt; 0,05 ili 0,01) u poređenju sa grupom sa jednostavnim T2DM. Serumski nivoi GMP1 su bili značajno viši kod pacijenata sa T2DM u odnosu na pacijente sa HTG. Binarnom logističkom regresionom analizom utvrđeno je da su serumski nivoi GMP1 (O R = 1,527, 95% CI 1,200-1,943; 95% CI 1,003-1,010) nezavisni faktor rizika za HTG kod T2DM pacijenata. Korelaciona analiza pokazala je slične rezultate, posebno kod pacijenata iz grupe T2DM +HTG. Višestrukom regresionom analizom je potvrđeno da, nakon kontrole faktora kao što su starost, pol, trajanje bolesti, BMI i HbA1c, viši nivoi GMP1 u krvi i dalje predstavljaju prediktor ili nezavisni faktor za T2DM komplikovan HTG (P&lt; 0,05).

**Zaključak:**

Serumski nivoi GMP1 su značajno poviseni kod pacijenata sa T2DM, posebno kod onih sa hipertrigliceridemijom, i nezavisno su pozitivno povezani sa nivoima triglicerida.

## Introduction

In recent years, the prevalence of diabetes worldwide has shown a continuous upward trend, becoming a significant health issue and imposing a heavy burden on our medical system and social economy. According to a literature report from 2024 [1], the proportion of adult diabetes patients is estimated to be as high as 12.8%. Type 2 diabetes mellitus (T2DM) is the most common type of diabetes, accounting for 90% of all types of diabetes [2]. The pathogenesis of T2DM is very complex and is related to various factors, such as genetics and the environment [3]. The rapid increase in the obese population is attributed mainly to an unhealthy lifestyle. Among them, the influence of a high-fat diet and a sedentary lifestyle is particularly prominent [4][5]. Obesity is a state caused by excessive accumulation of fat, especially triacylglycerol (TAG), in the body and is characterised by disordered fat metabolism. Hypertriglyceridemia (HTG) refers to a fasting serum TAG level of ≥1.7 mmol/L. There are complex associations between HTG and central obesity, metabolic syndrome, cardiovascular diseases, pancreatitis and diabetes [6][7][8][9]. Therefore, for patients with T2DM, an in-depth understanding of the mechanism of HTG occurrence and the implementation of effective management are crucial for improving their prognosis [10][11][12]. The succinylation of proteins is essential for maintaining metabolic homeostasis. Transcription factors can regulate various metabolic pathways through GMPylation, including those related to energy balance and lipid metabolism. Four GMP isomers have been discovered in mammalian cells: GMP1, SUMO2, SUMO3 and SUMO4 [13]. Among them, GMP1 is widely expressed in various tissues, including the kidneys, liver, adipose tissue and adrenal glands [14]. GMP1 is closely related to a variety of diseases, such as atherosclerosis, diabetes, neurodegenerative diseases, cancer and lipid metabolism disorders [15][16]. Studies have shown that a deficiency of SENP2 can lead to a reduction in lipid storage in adipose tissue, thereby triggering insulin resistance and ectopic fat accumulation, especially under high-fat diet conditions. These findings indicate that the degree of succinylation may be related to the increase in the serum TAG level [17]. Because of the important role that GMP1 plays in lipid metabolism and glucose metabolism, exploring its relationship with HTG in patients with T2DM is particularly important. This exploration not only helps us understand the function of GMP1 in metabolic regulation more comprehensively but also provides a new perspective for revealing the complex mechanisms of glucose and lipid metabolism.

Newly diagnosed T2DM patients who have not yet received treatment can help us eliminate influencing factors such as drug interference, thereby allowing us to study the relationship between GMP1 and HTG more accurately.

## Materials and methods

### Research subjects

From September 2023 to March 2025, the study included 239 patients newly diagnosed with type 2 diabetes at our institution. All of them underwent oral glucose tolerance tests by WHO diagnostic criteria and had not taken any drugs for the treatment of diabetes. Patients with liver failure, renal failure, malignant tumours or neurological disorders were excluded. There were 92 T2DM patients with HTG, with an average age of 52.43±11.84 years, and male patients accounted for 69.56%.

### Data collection

Two medical staff members conducted physical examinations on the patients. Blood samples were collected after a 10-hour fast. According to the »Prevention and Treatment of Adult Dyslipidemia Guidelines (2024 Edition),« a fasting triglyceride (TAG) level of <1.7 mmol/L is considered within the normal range, whereas a TAG level ≥1.7 mmol/L is classified as hypertriglyceridemia (HTG). The level of GMP1 was measured using a human GMP1 enzyme-linked immunosorbent assay (ELISA) kit (Orb562672, Biorbyt, UK).

### Laboratory testing methods

Collect 5 mL of fasting venous blood, let it stand at room temperature for 30 minutes, and then centrifuge at 3000 rpm for 10 minutes (centrifuge:) Eppendorf Centrifuge 5424R, Germany. The separated serum was aliquoted into EP tubes and frozen in a -80°C ultra-low temperature Freezer (Thermo Scientific ULT Freezer 902, USA) for testing.

All large-scale instruments are calibrated through daily quality control (Bole Bio-Rad quality control serum) and comply with ISO 15189 standards. ELISA experiments were conducted with duplicate Wells for detection, and the CV value was less than 10%. The blood lipid tests all met the requirements of the NCEP ATP guidelines.

### Detection of lipid status parameters

Total cholesterol (TC): Cholesterol oxidase method (Kit: Mindray TC Assay Kit, Item number: CHE0001), Instrument: Mindray BS-600 Biochemical Analyzer (China). Triglyceride (TG): GPO-PAP method (Kit: Liddman TG Kit, catalogue number: TR0100), with the same instrument as above. High-density lipoprotein cholesterol (HDL-C): Direct method (Kit: Shuishui Medical HDL-C EX, Item No. : 431-52501), Instrument: Hitachi 7180 Biochemical Analyzer (Japan). Low-density lipoprotein cholesterol (LDL-C): Direct selective clearance method (Kit: Jiuqiang Bio LDL-C Kit, catalogue number: L102), Tongmindray Instruments.

### Detection of other biomarkers

Serum GMP1 level: A double-antibody sandwich enzyme-linked immunosorbent assay (ELISA) was used. Kit: CUSABIO Human GMP1 ELISA Kit (Catalogue No.: CSB-EL027258HU), Manufacturer: Wuhan Huamei Biology, China. All detection steps were performed strictly according to the manufacturer's instructions. Microplate reader: BioTek Synergy H1 (USA), wavelength 450 nm.

Glycated haemoglobin (HbA1c): Measured by high-performance liquid chromatography (HPLC) using the Bio-Rad D-10 HbA1c Analysis System (BioRad Laboratories, USA).

Fasting plasma glucose (FPG): Determined using the hexokinase method. Kit: Roche Cobas c701 (Catalogue No.: 05168787); Instrument: Roche Cobas 8000 Automatic Biochemical Analyzer (Switzerland).

Fasting insulin (FINS): Measured by chemiluminescence. Kit: Siemens (Item No.: 03000064); Instrument: Siemens ADVIA Centaur XP (Germany).

### Statistical analysis

SPSS 25.0 was used to perform the analysis. x±s represents the normal distribution of the quantitative data. The 2 test was used to compare the rates of the two groups. To identify the factors impacting the serum GMP1 level, linear stepwise regression analysis was employed, and binary logistic regression was used to examine the components influencing HTG. A difference was considered statistically significant if P<0.05.

## Results

### Comparison of the baseline features of patients with T2DM with and without HTG

Compared with those of patients in the T2DM group without HTG, the BMI, WHR, diastolic blood pressure (DBP), HbA1c, and fasting blood glucose of patients in the T2DM group with HTG were relatively high (P<0.05). In contrast, the age and HDL levels were relatively low (P<0.05) (Table 1).

**Table 1:** Comparison of baseline characteristics between the T2DM with HTG group and the T2DM without HTG group.

Index	T2DM with HTG (n=92)	T2DM without HTG (n=147)	P value
Age, year	52.43±11.84	58.29±12.66	<0.001
Male, n (%)	64 (69.56)	84 (57.14)	0.054
BMI (kg·m^-2^)	26.34±4.00	25.04±3.20	0.006
WHR	0.94±0.06	0.92±0.05	0.024
SBP mmHg	139.42±17.71	137.32±16.18	0.349
DBP mmHg	87.83±11.89	83.53±11.48	0.006
HbA1c, n(%)	8.74±2.13	8.11±2.14	0.029
FBG (mmol·L^-1^)	9.36±3.07	8.33±2.61	0.006
HDL (mmol·L^-1^)	1.11±0.21	1.31±0.34	<0.001
LDL (mmol·L-^-1^)	3.43±1.02	3.17±0.99	0.055
TC (mmol·L^-1^)	5.62±1.13	5.05±1.17	<0.001
TAG (mmol·L^-1^)	2.37 (2.00, 3.48)	1.25 (0.88, 1.48)	<0.001
Insulin (pmol·L^-1^)	57.00 (42.45, 94.45)	50.88 (34.52, 78.73)	0.028
Scr (μmol·L^-1^)	64.09±15.16	61.63±16.03	0.243
ALT (U·L^-1^)	32.00 (20.00, 50.25)	24.00 (17.00, 34.25)	0.100
AST (U·L^-1^)	24.50 (20.00, 33.00)	21.50 (17.75, 26.00)	0.327
UA (μmol·L^-1^)	375.76±94.36	325.33±87.76	<0.001
Smoking, n(%)	31 (33.69)	33 (22.45)	0.06
Drinking, n(%)	26 (28.26)	33 (22.45)	0.325

### Comparison of serum GMP1 levels

Patients with T2DM paired with HTG had considerably higher blood GMP1 levels than those with T2DM alone.

### Factors influencing HTG

Binary logistic regression revealed that UA, HbA1c, and GMP1 were independent risk factors for HTG after adjustments were made (Table 2).

**Table 2:** Influencing factors of HTG by Logistic regression analysis.

Independent variable	β	Exp (β)	95%CI	P value
GMP1	0.423	1.527	1.200-1.943	<0.001
HbA1c	0.184	1.202	1.038-1.391	0.010
UA	0.006	1.006	1.003-1.010	<0.001

### The influence of the serum GMP1 level on HTG

The patients were split into four quartiles according to their serum GMP1 levels (<691 pg/mL, 692-887 pg/mL, 888-1,187 pg/mL, and ≥1,188 pg/mL) (Table 3). Taking the lowest quartile as the reference, i.e., Scr, ALT and AST, the risk of HTG in group Q4 significantly increased and was 2.707 times greater than that in group Q1 (95% CI 1.231-5.951).

**Table 3:** Impact of serum GMP1 levels on HTG.

GMP1 quartile	n/total	Crude OR (95%CI)	Model 1 OR (95%CI)	Model 2 OR (95%CI)
Q1 (<691 pg·mL^-1^)	15/59	1	1	1
Q2 (692-887 pg·mL^-1^)	18/60	1.257 (0.562~2.812)	1.270 (0.566~2.850)	1.319 (0.584~2.976)
Q3 (888-1 187 pg·mL^-1^)	27/58	2.550 (1.170~5.578)	2.440 (1.112~5.355)	2.419 (1.097~5.332)
Q4 (>1 188 pg·mL^-1^)	32/62	3.129 (1.450~6.752)	2.886 (1.322~6.298)	2.707(1.231 ~5.951)
P value		0.008	0.010	0.010

### Factors influencing serum GMP1 levels

WHR, TAG, and Scr were found to be independent risk variables for an increase in the serum GMP1 level (P<0.05) via multiple linear regression analysis. Compared with that in men, the serum GMP1 level in women increased more significantly (Table 4). This result shows that there is a mutual relationship between the blood TAG level and the serum GMP1 level.

**Table 4:** Influencing factors of serum GMP1 levels by multiple linear regression analysis.

Independent variable	Standardised β	t	P value
Male	-0.218	-3.438	0.001
WHR	0.165	2.538	0.012
TAG	0.151	2.324	0.021
Scr	0.205	3.186	0.002

## Discussion

Compared with those of patients without HTG, the serum GMP1 levels of patients with HTG were significantly greater. Stratified based on quartiles of serum GMP1 levels. The results of this study indicate that in newly diagnosed T2DM patients, there is a mutual influence relationship between serum GMP1 levels and HTG.

There is a close connection between HTG and patients with T2DM, and HTG is a common dyslipidemia in patients with T2DM [18]. This connection stems mainly from the pathogenesis and progression mechanism of T2DM, among which metabolic factors such as insulin resistance (IR) and central obesity play key roles. IR is a significant feature of T2DM. Research [19] indicates that there is a positive correlation between IR and HTG, and the TAG level is also regarded as a good indicator of IR. Furthermore, HTG has been proven to be an essential trigger of IR [20]. The relationship between T2DM and HTG is not a simple causal relationship but manifests as a complex relationship of mutual influence. In many cases, these two factors coexist and may intensify each other, forming a vicious cycle that is difficult to break.

As a rapid and efficient way to regulate protein activity, reversible posttranslational modification plays a crucial role in regulating life activities. Sumoylation has multiple functions and is involved in processes such as transcription, DNA repair, macromolecule assembly, protein homeostasis, transport and signal transduction [11][21]. Sumoylation plays a key role in maintaining metabolic homeostasis. It enables various metabolic activities to proceed in an orderly and efficient manner by influencing the functions of key enzymes and the activities of transcription factors in metabolic pathways, as well as promoting the segmentation of intracellular metabolic processes [12]. In addition, GMP elongation also plays a crucial role in regulating nutritional and metabolic sensing mechanisms. The synergistic effect of these functions makes succinylation modification an indispensable regulatory factor for maintaining cellular metabolic homeostasis. Therefore, in-depth research on the specific mechanism of succinylation in metabolic homeostasis is expected to reveal the pathogenesis of more metabolism-related diseases.

Four GMP proteins, namely, GMP1, SUMO2, SUMO3 and SUMO4, have been discovered in vertebrates. Among them, GMP1 is a protein with a relative molecular mass of 12,000, composed of 101 amino acids, widely distributed in the body, and functions mainly through succinylation [22]. Previous studies [23][24][25] using human islet cells for single-cell RNA sequencing have shown that the expression level of GMP1 is consistent across all types of islet endocrine cells [26]. Some studies have also demonstrated that deficiency or overexpression of the 62-binding enzyme Ubc9 in pancreatic islet b cells can lead to impaired antioxidant capacity, reduced insulin content, and loss of β-cell mass [27]. SuMOylation plays a complex role in the regulation of blood glucose in the body. Under normal conditions, the concentration of GMP1 maintains a dynamic balance within a specific range. Disruption of this balance may lead to disorders in the body's functions. GMP1 also plays a crucial role in lipid metabolism. It finely regulates lipid metabolism through the modification of key enzymes and influences lipid metabolism regulatory factors [28].

Peroxisome proliferator-activated receptor (PPAR ) is regarded as the primary regulatory factor of adipogenesis [29]. PPAR has significant effects on adipogenesis, systemic lipid metabolism and insulin sensitivity [30]. Studies have shown that GMP1 can perform succinylation modifications on PPAR, thereby increasing lipid production [31]. PPAR is expressed mainly in adipocytes, and its primary functions are to improve the insulin sensitivity of cells and promote the uptake of fatty acids and the maturation of adipocytes. GMP1 can inhibit the activation of PPAR. The inhibition or decreased expression of PPAR leads to decreased insulin sensitivity, reduced fatty acid uptake and blocked adipocyte maturation. The absence or insufficiency of adipocytes, as the main storage sites of TAG, can lead to the release of a large amount of TAG into the bloodstream, thereby causing HTG. Thus, GMP1 can regulate lipid metabolism in the body through PPAR. Forkhead box protein A2 (FOXA2) can increase the synthesis and secretion of TAG, while sumoylation can increase the transcriptional activity of FOXA2.

Thus, by activating FOXA2, succinylation can promote the synthesis and secretion of TAG [32]. The succinylation of SREBPs can reduce the biosynthesis and accumulation of lipids. GMP1 regulates lipid triglycmetabolism by regulating different target proteins and modifying different transcription factors, thereby establishing a complex lipid regulatory network. At present, there are no studies on the differences in the expression level of GMP1 between different sexes. However, studies have shown that the estradiol-estrogen receptor signalling pathway can upregulate the transcription of GMP1, which may explain why the serum GMP1 level in women is higher than that in men. Studies have shown that the level of GMP1 in the serum of patients with Alzheimer's disease is elevated. However, no research on the mechanism by which GMP1 enters the bloodstream has been conducted. Possible mechanisms include the release of GMP1 into the blood after cell death and disruption.

## Conclusion

Exploring the correlation between the level of GMP1 in the serum of newly diagnosed T2DM patients and HTG can provide a new perspective for the mechanistic study of glucose and lipid metabolism disorders. In-depth research on SUMOylation is expected to provide more effective strategies for the treatment and prevention of metabolic diseases such as T2DM and HTG.

## Dodatak

### Conflict of interest statement

All the authors declare that they have no conflict of interest in this work.
